# An Analysis of the Use of Systemic Antifungals (Fluconazole, Itraconazole, and Terbinafine) in Galicia, Spain, between 2019 and 2022

**DOI:** 10.3390/diseases12010022

**Published:** 2024-01-12

**Authors:** Severo Vázquez-Prieto, Antonio Vaamonde, Esperanza Paniagua

**Affiliations:** 1 Laboratorio de Parasitología, Departamento de Microbiología y Parasitología, Facultad de Farmacia, Universidad de Santiago de Compostela, Campus Vida, 15782 Santiago de Compostela, Spain; mesperanza.paniagua@usc.es; 2 Núcleo de Investigación en Ciencias de la Salud, Universidad Adventista de Chile, Chillán 3780000, Chile; 3 Departamento de Estadística e Investigación Operativa, Facultad de Ciencias Económicas y Empresariales, Universidad de Vigo, 36310 Vigo, Spain; vaamonde@uvigo.gal; 4 Instituto de Investigación en Análisis Químicos y Biológicos (IAQBUS), Universidad de Santiago de Compostela, 15782 Santiago de Compostela, Spain

**Keywords:** drug utilization study, defined daily dose, fluconazole, itraconazole, terbinafine

## Abstract

In the present work, we examined the consumption of systemic antifungals (fluconazole, itraconazole, and terbinafine) in outpatients in the four provinces of Galicia, Spain, between 2019 and 2022. We also described the variability in the use of these types of drugs between these provinces. In addition, we detected any deviation in consumption at a seasonal level and analyzed possible changes during the study period. A descriptive, cross-sectional, and retrospective study of the use of antifungals, expressed in terms of a defined daily dose per 1000 inhabitants per day, was carried out. The results obtained revealed statistically significant differences between provinces and by the active principle consumed in the four Galician provinces (*p* < 0.001), which can be explained by multiple factors. This study also revealed that there was stable consumption during the study period, with no significant seasonal differences observed. This study represents a contribution to the knowledge about the consumption of antifungals for systemic use in Galicia and serves as a basis for subsequent studies. This will allow us to understand the consumption patterns of these types of drugs and, ultimately, will help to establish stewardship strategies and prevent the development of resistance.

## 1. Introduction

Fungi are a group of organisms classified as a kingdom within the Eukarya domain; they are widely distributed in nature and can live on human organisms as saprophytes or parasites. Fungal infections, also known as mycoses, are traditionally demarcated as superficial (when they are limited to the skin, hair, nails, and mucous membranes), subcutaneous (when a granulomatous response of the skin and subcutaneous tissue occurs at the inoculation site), and systemic (when the infection is deep and affects internal organs) [1]. In recent decades, there has been a notable increase in their incidence due to the increase in immunosuppressed patients with cancer or undergoing solid organ transplantation, the AIDS and type 2 diabetes epidemic, and the use of broad-spectrum antimicrobials and drugs that alter the natural defense mechanisms [2,3].

Three systemic antifungals are available for dispensing in Spanish community pharmacies, for which a medical prescription is necessary. These include the imidazole derivatives fluconazole and itraconazole. There are slight differences between them in the spectrum of action, but the most important distinction is that fluconazole is highly soluble in water, while itraconazole is lipophilic. Thus, fluconazole is administered orally or parenterally; food and gastric pH do not influence its absorption; its elimination is mainly renal; and it presents low binding to plasmatic proteins and high concentrations in urine, cerebrospinal fluid, and intraocular space. In contrast, itraconazole is only administered orally; its absorption is increased when administered with food and decreased by antacids and H2 antagonists; its elimination is fundamentally hepatic and presents a high binding to plasmatic proteins; and it is concentrated in fatty tissues, skin, and nails and does not pass into the cerebrospinal fluid. The values reached in the tissues by both drugs remain at high concentrations once their administration is completed. These pharmacokinetics allow the use of pulsatile patterns (once a week or one week a month), maintaining the effectiveness of the drugs and reducing their side effects. The other antifungal available is terbinafine. After oral absorption, it is concentrated in the lipophilic stratum corneum, reaching high concentrations in hair follicles, hair, and oily skin. It is rapidly metabolized and excreted mainly in the urine. Terbinafine is effective almost exclusively in dermatophyte infections, and its main applications by systemic administration are for ringworms of the scalp, onychomycosis, and cases of *tinea pedis* with plantar involvement [4].

The emergence of resistant fungi constitutes a major global health problem. The factors that lead to it are diverse and heterogeneous [5], highlighting the excessive use and misuse of antifungal agents [6,7,8,9]. Thus, the increased consumption of these types of drugs results in an increased selection pressure on fungal strains and may lead to an increasing number of resistant pathogens [10]. Therefore, monitoring the consumption of antifungals is essential to understanding the patterns of their use and thus implementing measures that favor the proper use of these types of drugs to safeguard public health [11].

In the literature, there are publications on the consumption of antifungals by hospitalized patients [12,13,14] and critically ill patients [15,16,17,18] or within general studies of antimicrobial use [19,20,21], but there are few published studies on the patterns of use in outpatient settings [22,23,24]. Available data on the consumption of systemic antifungals in Spain are scarce and often limited to the hospital sector or a specific hospital unit [25,26,27]. In addition, to the best of our knowledge, there are no studies on the consumption of these substances in Galicia, the fifth-largest autonomous community with the largest population and surface area in Spain. In the present work, we first examined the consumption of systemic antifungals in outpatients in the four provinces of Galicia between 2019 and 2022 and described the variability in the consumption of these types of drugs between these provinces. Secondly, we detected any deviation in consumption at a seasonal level and analyzed possible changes during the study period. This study was conducted using the anatomic therapeutic chemical (ATC) and defined daily dose (*DDD*) methodologies, which are internationally accepted for measuring drug use within and between populations [28,29].

## 2. Materials and Methods

We carried out a descriptive, cross-sectional, and retrospective study in Galicia, an autonomous community of Spain located in the northwest of the country that occupies a total area of 29,574 km^2^ and is territorially structured into four provinces: Pontevedra, Ourense, Lugo, and A Coruña. Consumption data of the subgroups J02AC (fluconazole and itraconazole) and D01BA (terbinafine) of the ATC code classification were obtained from the prescription billing information system charged to the Galician Health Service (SERGAS) between January 2019 and December 2022. The consumption data were expressed in *DDD*s per 1000 inhabitants per day (*DID*) according to the following formula:

$$DID=\frac{nºDDD\times 1000}{Population\times t}$$

where *nº DDD* represents the total number of *DDD*s, *t* is the number of days in a month, and *population* is the total number of people living in each province for the year the drug was dispensed. The annual population data were obtained from a publicly accessible demographic database of the Galician Institute of Statistics [30]. The *DDD* is a technical unit of measurement defined by the WHO Collaborating Centre for Drug Statistics and Methodology as the usual maintenance dose in adults for its main indication per day. The use of *DID* makes it possible to compare the use of drugs in different regions and over time without being influenced by market differences in terms of number of dosage forms or concentration of active principles in packages. As such, *DID* is an indicator of the prevalence of use of a certain drug in the population [31].

For the processing of information, a data set was created in the Excel program. Statistical analysis was performed with the free statistical program R version 4.0.3. The comparison of consumption between provinces and between active principles was carried out using the non-parametric Kruskal–Wallis test. A *p*-value of < 0.05 was considered statistically significant. The results were illustrated using box plots, where the median (the thick horizontal line), the Q1 and Q3 quartiles (the edges of the box), and the maximum and minimum values (the ends of the whiskers) were represented.

## 3. Results and Discussion

Nearly one billion people worldwide are estimated to suffer from fungal infections of the skin, nails, and hair, and more than 150 million suffer from serious fungal diseases that have a significant impact on their lives. Drug-resistant fungal infections are becoming more common, complicating an already difficult therapeutic situation [24,32,33]. Here, we carried out, to the best of our knowledge, the first drug utilization study focused on the consumption of systemic antifungals in Galicia based on data obtained from community pharmacies. A drug utilization study includes an analysis of the commercialization, distribution, prescription, and use of drugs in society, with particular attention to the results of their medical, social, and economic consequences [34]. Through the use of specific quantitative parameters, these studies can provide an accurate estimate of the volume of the population treated daily with a typical dose of a given drug. They can also allow comparisons of consumption in various geographic locations or in the same region over time, regardless of variations in prices or changes in the way a drug is presented [35,36,37].

**Figure 1:**
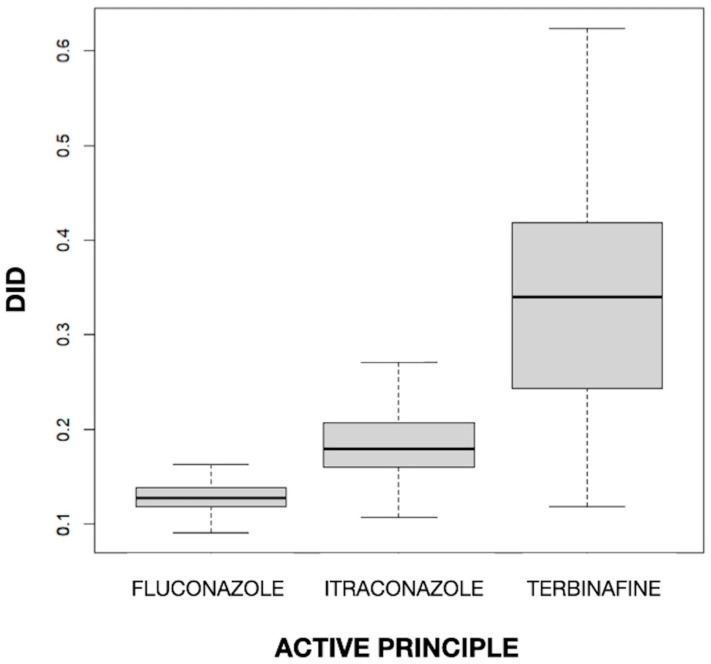
Values of the defined daily dose per 1000 inhabitants per day (*DID*) by active principle.

The mean values of *DID* for fluconazole, itraconazole, and terbinafine were 0.129, 0.187, and 0.336, respectively. The difference in the mean *DID* between the three drugs was statistically significant (*p* < 0.001). In Figure 1, a clear difference between drugs can be observed, with a higher value (both in the median and in the box as a whole) for terbinafine and a lower value for fluconazole and itraconazole, and also a different dispersion, with greater variability for terbinafine and much less variability for the imidazole derivatives. The fact that terbinafine was the most used antifungal is in line with the finding of Baştuğ et al. [38] in Turkey, where, with a rate of 66.6% in outpatient care, it was the most used agent, followed by itraconazole (20.8%) and fluconazole (11.9%). Terbinafine, itraconazole, and fluconazole were found to have a mean *DID* level of 1.057, 0.329, and 0.188, respectively, among outpatients. On the other hand, terbinafine was the most commonly used antifungal agent and the second most commonly used antifungal agent in high-income and middle-income countries, respectively [39]. This is congruent with the high prevalence of superficial nail and skin fungal infections and the importance of using this drug in their treatment [10,32].

However, fluconazole was the most prescribed antifungal in a cross-sectional study carried out in outpatients in a Colombian population, which was also consistent with findings in the United States, Australia, and Tanzania [22,23,40]. Similarly, these studies of outpatients were consistent with what was found in studies of hospitalized patients [24,25,26]. On the other hand, itraconazole (0.32 *DID*), terbinafine (0.30 *DID*), and fluconazole (0.23 *DID*) were the most commonly used substances in middle- and high-income countries in 2018 [39]. The fact that fluconazole and itraconazole are the agents of choice for the treatment of vulvovaginal candidiasis, which affects almost 75% of women at least once in their lives, may contribute to a substantial proportion of their use [39,41].

**Figure 2:**
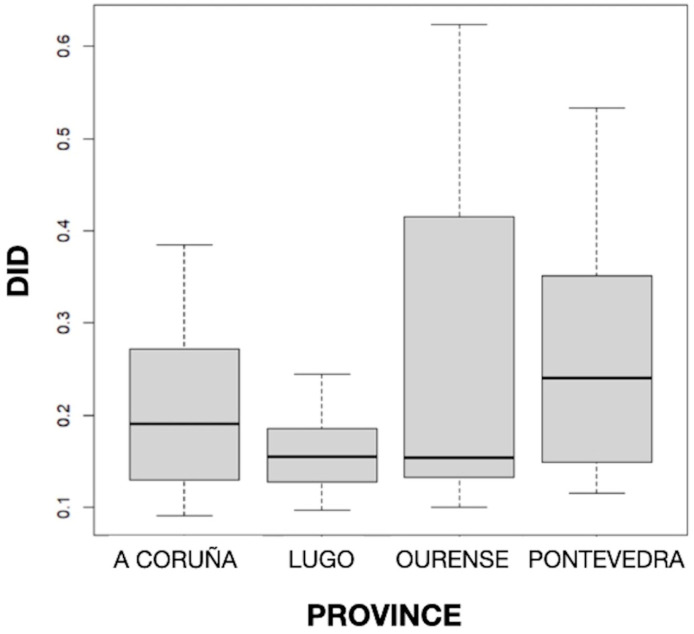
Distribution of defined daily dose per 1000 inhabitants per day (*DID*) by province.

The distribution of *DID* by provinces can be seen in Figure 2, and the mean values of *DID* by the active principle and province can be seen in Table 1. The results obtained showed statistically significant differences (*p* < 0.001) in the mean *DID* values between the four Galician provinces, with the highest and lowest consumption found in Pontevedra (0.255) and Lugo (0.162), respectively. But no particular geographical pattern was noted.

The consumption rates of systemic antifungals in Galicia are somewhat similar to those in Spain. Thus, the consumption of terbinafine, fluconazole, and itraconazole in the Spanish community sector (primary care) in 2021 was 0.21, 0.11, and 0.12 *DDD*/1000 inhabitants/day, respectively. Large variability in the consumption of these drugs was observed in community settings within European Union and European Economic Area countries, ranging from 0.00 to 2.60 for terbinafine, 0.08 to 0.86 for fluconazole, and 0.01 to 1.58 for itraconazole [42]. Disparities in the pharmaceutical market, healthcare systems, social and cultural aspects, regulatory frameworks, and available resources may have an impact on the consumption pattern of systemic antifungals between European countries [11,43].

The differences found in the consumption of antifungals by province may be explained by several factors. For example, climate may influence the incidence and type of fungal infections, with humid weather favoring fungal growth, sunny and windy weather favoring the spread of spores, and snow considerably reducing both events [44]. Geographic variability could also be related to prescriber preferences, compliance with clinical practice guidelines, drug resistance and susceptibility patterns, general health status, and social determinants of health in each province [22,24,39,45,46]. The Spanish National Plan Against Antibiotic Resistance (PRAN) was launched in 2014. It is structured into six strategic lines (surveillance, control, prevention, research, training, and communication) and aims to raise public awareness about the need for prudent use of antimicrobials. However, only in the latest strategic plan published, which establishes priority actions for the years 2022–2024, is the surveillance of antifungals in human health and the control of resistant fungi proposed [47]. Although Galicia has been working for years to control antimicrobial resistance, no entity has been established that allows for common monitoring of initiatives and problems, coordinating messages to the population, and monitoring progress to achieve predefined objectives. However, a Galician commission has been created with the aim of addressing the aforementioned issues, identifying those that must be transferred to higher levels for decision making, and formulating those that require legislative action [48].

**Figure 3:**
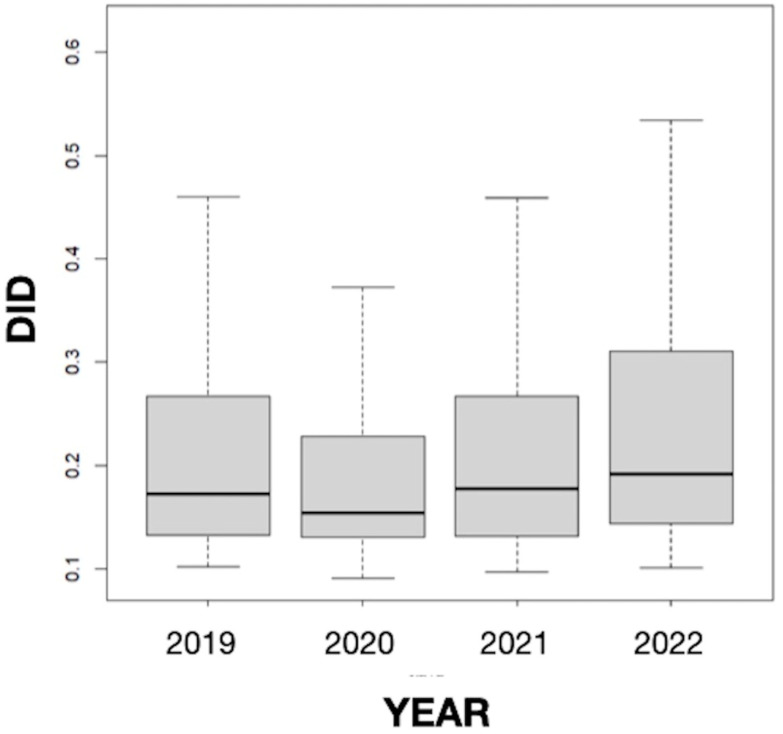
Annual evolution of the defined daily dose per 1000 inhabitants per day (*DID*) from 2019 to 2022 in Galicia (Spain).

The results obtained in our study did not suggest seasonality since the differences in the mean values of the *DID* variable between months were not statistically significant, but they revealed a somewhat stable consumption of antifungals in Galicia throughout the study period, which includes the COVID-19 pandemic (Figure 3). This result coincides with the data for Spain as a whole (0.235 in 2019, 0.203 in 2020, and 0.230 in 2021) [49] but differs from those found in other countries. Thus, Pathadka et al. [39] reported, using pharmaceutical sales data from wholesalers, that systemic antifungal consumption increased from 0.50 to 0.92 *DDD*/1000 inhabitants/day between 2008 and 2018 in 65 middle- and high-income countries. Similarly, Rahme et al. [11], based on the volume of antifungals sold in community pharmacies, described an increase of 18.64% in the consumption of this type of medicine between 2004 and 2018 in Lebanon. Our finding also differs from other previous works carried out in Spain in the hospital setting. For example, a study conducted in Catalonia between 2008 and 2013 showed a sustained upward trend (20.5%; *p* = 0.066) in the overall consumption of systemic antifungals in all acute care hospitals studied. Furthermore, the consumption of antifungals was higher in the intensive care units (ICUs) than in the medical and surgical services, increasing by 12.4% (*p* = 0.019) [25]. In an observational prospective study of the systemic use of antifungal agents in patients admitted to Spanish ICUs from 2006 to 2010, Olachea et al. [26] found an increase in the global consumption of antifungals from 2006 to 2008, with a stabilization in the following two years. Similarly, the antifungals for systemic use prescribed at the University Clinic Hospital of Valladolid during the years 2009–2013 showed an upward trend in *DDD*s in the study period, with pronounced oscillations that were statistically significant in most cases [27]. The consumption pattern observed in our study could be related to the absence of variations in drug resistance, prescribing practices, and the health system at the community level in recent years.

The main strength of this study is that, to our knowledge, it is the first to address the consumption of systemic antifungals in Galicia based on data obtained from prescriptions dispensed in community pharmacies. This work provides a basic description of the use of these substances in the region, a preliminary step to better understand where to focus stewardship efforts.

This study has some limitations. First, *DDD* does not necessarily correspond to the prescribed daily dose and/or the amount of medicine consumed by the patient in practice [37]. Second, the use of antifungal agents with a private or mutual medical prescription, as well as those dispensed without a medical prescription, was excluded from this study; however, the number of these dispensations is estimated to be low (Carmen Goicoa Gago pharmacy, personal communication). Third, indication information is missing. Fourth, the nature of the data could lead to an overestimation of drugs used for long-term treatments, such as terbinafine for onychomycosis [22]. Finally, there are few studies in the literature that use a similar methodology to analyze the outpatient consumption of the three drugs considered here with which the results can be compared. In addition, it should be noted that there may be significant differences in antifungal prescribing practices between countries or regions due to factors such as susceptibility to antifungal agents, infection control practices, burden of immunocompromised individuals, and accessibility to resources. These differences may affect observed consumption patterns and limit comparisons [39].

In conclusion, the present study showed statistically significant differences between provinces and by the active principles fluconazole, itraconazole, and terbinafine consumed in the four Galician provinces, which could be explained by differences in multiple aspects between provinces, such as climatic conditions, prescriber preferences, compliance with clinical practice guidelines, drug resistance and susceptibility patterns, general health status, or social determinants of health. Although our study revealed that the use of antifungals in Galicia remains at relatively low levels within the European range and that there was a stable consumption during the period 2019–2022, without significant seasonal differences being observed, it is advisable to continue improving antimicrobial surveillance and analyses, as well as the coordination of health policies, at the regional and national levels to strengthen the effectiveness of public health actions aimed at promoting the rational use of antifungals. This study represents a contribution to the knowledge about the consumption of antifungals for systemic use in Galicia and serves as a basis for subsequent studies that include, for example, hospital and outpatient prescription, information on indications, as well as dose and duration of treatment. This will allow us to understand the consumption patterns of these types of drugs and will, ultimately, help to establish antifungal stewardship strategies and prevent the development of resistance.

## Figures and Tables

**Table 1:** Mean values of the defined daily dose per 1000 inhabitants per day (*DID*) by active principle and province.

Province	Fluconazole	Itraconazole	Terbinafine
A CORUÑA	0.121	0.186	0.3
LUGO	0.122	0.172	0.192
OURENSE	0.131	0.155	0.467
PONTEVEDRA	0.141	0.236	0.387

## Data Availability

The raw data supporting the conclusions of this article will be made available by the authors on request.
